# Characterization of the Vaginal DNA Virome in Health and Dysbiosis

**DOI:** 10.3390/v12101143

**Published:** 2020-10-09

**Authors:** Rasmus Riemer Jakobsen, Thor Haahr, Peter Humaidan, Jørgen Skov Jensen, Witold Piotr Kot, Josue Leonardo Castro-Mejia, Ling Deng, Thomas Dyrmann Leser, Dennis Sandris Nielsen

**Affiliations:** 1 Section of Microbiology and Fermentation, Department of Food Science, Faculty of Science, University of Copenhagen, DK-1958 Copenhagen, Denmark; jcame@food.ku.dk (J.L.C.-M.); lingdeng@food.ku.dk (L.D.); dn@food.ku.dk (D.S.N.); 2 Department of Clinical Medicine, Aarhus University, DK-8200 Aarhus, Denmark; thohaa@rm.dk (T.H.); peter.humaidan@midt.rm.dk (P.H.); 3 The Fertility Clinic Skive, Skive Regional Hospital, DK-7800 Skive, Denmark; 4 Research Unit for Reproductive Microbiology, Statens Serum Institut, DK-2300 Copenhagen, Denmark; JSJ@ssi.dk; 5 Department of Environmental Sciences, Aarhus University, Risø, 4000 Roskilde, Denmark; wk@envs.au.dk; 6 Human Health, Innovation, Chr. Hansen A/S, 10-12 Boege Allé, DK-2970 Hoersholm, Denmark; DKTDL@chr-hansen.com

**Keywords:** vaginal microbiome, vaginal virome, bacteriophages, bacterial vaginosis, dysbiosis

## Abstract

Bacterial vaginosis (BV) is characterized by a reduction in *Lactobacillus (L.)* spp. abundance and increased abundance of facultative anaerobes, such as *Gardnerella* spp. BV aetiology is not fully understood; however, bacteriophages could play a pivotal role in the perturbation of the vaginal bacterial community. We investigated the vaginal viral community, including bacteriophages and the association to the bacterial community and BV-status. Vaginal samples from 48 patients undergoing IVF treatment for non-female factor infertility were subjected to metagenomic sequencing of purified virus-like particles. The vaginal viral community was characterized and correlated with the BV-status by Nugent score, bacterial community, structure, and the presence of key vaginal bacterial species. The majority of identified vaginal viruses belonged to the class of double-stranded DNA bacteriophages, with eukaryotic viruses constituting 4% of the total reads. Clear links between the viral community composition and BV (*q* = 0.006, *R* = 0.26) as well as the presence of *L. crispatus* (*q* = 0.001, *R* = 0.43), *L. iners*, *Gardnerella* spp., and *Atopobium vaginae* were found (*q* < 0.002, *R* > 0.15). The eukaryotic viral community also correlated with BV-status (*q* = 0.018, *R* = 0.20). In conclusion, the vaginal virome was clearly linked with bacterial community structure and BV-status.

## 1. Introduction

The vaginal microbiota (VMB) refers to the microorganisms inhabiting the vagina. Until now, the majority of studies have focused on bacteria and fungi, whereas little is known about the viral community. When dominated by *Lactobacillus* spp., especially *L. crispatus*, the VMB has a protective role in preventing bacterial vaginosis, yeast infections, sexually transmitted infections (STIs) and urinary tract infections [1,2]. However many asymptomatic women have more diverse VMB [3,4], and the degree of *Lactobacillus* dominance appears to be less prevalent in communities of non-European ancestry [5,6]. Bacterial vaginosis (BV) is the most common dysbiosis of the VMB, affecting 10–30% of reproductive age women in developed nations [7]. BV is characterized by a reduction of vaginal *Lactobacillus* abundance and an increase in the relative abundance of other facultative anaerobic bacteria [8]. BV and other forms of VMB dysbiosis have been reported to be important risk factor for STI acquisition [9] and adverse reproductive outcomes [10].

Most healthy women have a stable and relatively simple vaginal bacterial community dominated by one single *Lactobacillus* spp. However, it is well-known that perturbations of the VMB occurs during menses and antibiotic treatment [11], and are affected by the number of sexual partners [12] while being protected by male circumcision [13]. Although *Gardnerella* spp. is generally accepted as one of the key bacteria involved in BV pathogenesis [14], the exact aetiology of BV is still undergoing further investigation.

The total collection of vaginal viruses (the vaginal “virome”), has only been sparsely investigated; however, there is increasing evidence that bacteriophages (or “phages”) are a factor in certain diseases related to gut microbiome dysbiosis [15]. Phages adhere to and are significantly enriched in mucosal surfaces, possibly providing what has been termed “non-host derived immunity” against infection [16,17].

Phages are viruses that target bacteria in a host-specific manner. Phages use one of two fundamentally different methods of replication. Lytic phages infect the bacterial cell, direct the biosynthesis to new phages, and then lyse the cell to release the new phages. Temperate phages are also able to enter the lytic cycle, but additionally—and in contrast to lytic phages—they are able to replicate through the lysogenic cycle. In this cycle, the phage inserts its genetic material into the prokaryote genome as a prophage, which lies latent and is transmitted vertically during bacterial replication until activated, whereupon the lytic cycle commences [18]. A possible mechanism underlying VMB dysbiosis could be prophage induction causing community shifts [19]. In support of this hypothesis, research has been shown that VMB-related *Lactobacillus* spp. contain prophages that are activated by environmental stressors, such as smoking [20].

Investigation of a virome using metagenomic sequencing can be performed with or without the purification of virus-like particles (VLPs). The purification of VLPs has the advantage of removing the majority of bacterial and host DNA allowing deeper sequencing of viral DNA, with the disadvantage that some viral particles may be lost during purification [21]. Alternatively, viral reads can be identified and recovered from metagenomes by matching to known viral genomes, or using a variety of computational tools [22]. This has the disadvantage that the majority of reads from full metagenome sequencing will be from bacterial genomes due to their much larger genome size [23], and significant bias toward the detection of viral families more well-characterized in genome databases [24].

A recent study investigated the possible links between vaginal dysbiosis and the risk of HIV acquisition in South African women. The study also investigated the vaginal virome, but reported no distinct viral community structures within their cohort [25]. However, these findings should be interpreted with caution, as the experimental protocol was based on the filtration of VLPs from low volumes of cervicovaginal lavage (CVL) without any up-concentration steps, yielding low input for downstream analysis. The bioinformatics analysis was based on read mapping exclusively to NCBI viral sequences, therefore, excluding uncharacterized viruses or phages incorporated into known bacterial genomes as prophages. Another recent study used bioinformatically extracted viral reads from vaginal metagenomes [26], noting the presence of the eukaryotic virus families Herpesviridae and Partitiviridae, but without analysing the bacteriophage community in detail. Finally, a study, based on the targeted purification of vaginal eukaryotic viral nucleic acids using biotinylated probes targeting all vertebrate viruses prior to high throughput sequencing, found that increased vaginal eukaryotic viral richness was significantly associated with preterm birth [27].

Here, we report a cross-sectional investigation of the vaginal virome of 48 healthy Danish women submitted to IVF treatment of non-female factor infertility. Using metagenome sequencing of highly purified VLPs from vaginal swabs, we aimed to elucidate both the overall composition and variability of the vaginal bacteriophages and eukaryotic viral component and to explore associations with the vaginal bacterial community state types considered either healthy or dysbiotic.

## 2. Materials and Methods

### 2.1. Patients and Samples

This study is a sub-study of a larger comparative clinical study [28] that developed a novel molecular based diagnosis of abnormal vaginal microbiota reporting poor reproductive outcomes in IVF treatment [29]. All patients signed written informed consent prior to enrollment. The present study involved 48 women admitted to IVF treatment due to either a male factor, being single, or being lesbian (Table 1). Patients with known female reproductive disorders were excluded. This was done to focus the study on establishing a baseline of the vaginal virome of healthy, reproductive-age women. In brief, patients were recruited at two IVF centers in Denmark and they were prospectively included at their first consultation before the initiation of their IVF treatment [28]. Samples were taken from the posterior fornix during speculum examination prior to treatment. One sample was used for Gram staining and Nugent scoring [30]. The second sample was taken for molecular analyses using a 1 mL Copan ESwab™ (Copan Italia, Brescia, Italy) and frozen at −80 °C until further use.

### 2.2. Characterisation of the Bacterial Component of the VMB

The bacterial component of the VMB was characterised using a combination of 16S rRNA gene amplicon profiling and quantitative (q) PCR analyses for *G. vaginalis*, *A. vaginae*, *L. crispatus*, *L. jensenii*, *L. gasseri,* and *L. iners* [31]. The presence of abnormal vaginal microbiota (AVM) was determined using the presence/absence of key vaginal bacterial species (*G. vaginalis, A. vaginae, L. crispatus, L. jensenii, L. gasseri, and L. iners),* which was measured by qPCR to classify the vaginal microbiota as described elsewhere [31]. The clustering of samples into community states (CSTs) was performed using linkage clustering based on the bacterial composition and abundance in accordance with Ravel et al. [2]. These data were previously published [28] and were made available for the present study.

### 2.3. Virus Like Particles (VLP) Purification, Viral DNA Extraction, and Sequencing

We diluted 1 mL of Copan ESwab buffer containing vaginal material in 5 mL of Saline Magnesium (SM)-buffer (200 mM NaCl, 50 mM Tris HCl, 8 mM MgSO4·7H2O, pH 7.4) and filtered through 0.45 µm Minisart^®^ High Flow Polyethersulfone (PES) syringe filter (Sartorius, Göttingen, Germany). The filtrate was concentrated, and particles below 50 kDa were removed using Centriprep^®^ Ultracel^®^ YM-50-kDa filter units (Millipore, Burlington, MA, USA) centrifuged at 1500× *g* at 25 °C until approximately 300 µL was left in the outer tube. This was defined as the concentrated virome. The 50 kDa filter from the inner tube was removed and added to the concentrated virome and stored for at 4 °C until nucleic acid extraction. We treated 140 µL of virome with 2.5 units of Pierce™ Universal Nuclease (ThermoFisher Scientific, Waltham, MA, USA) for 3 min, to remove free DNA/RNA molecules. The nucleases were inactivated by 560 µL AVL buffer from the QIAamp^®^ Viral RNA Mini kit (Qiagen, Hilden, Germany). 

For viral DNA extraction, the NetoVIR procotol [32] was followed from step 11–27, (with the elution buffer (AVE) volume adjusted to 30 µL). The samples were stored at −80 °C until viral genome amplification. The Illustra Ready-To-Go GenomiPhi V3 DNA Amplification Kit (GE Healthcare Life Sciences, Amersham, UK) was used for viral genome amplification following the instructions of the manufacturer, but with the DNA amplification decreased to 60 min (rather than 90 min) to decrease the amplification bias [33]. We used 10 units of Genomic DNA Clean & Concentrator™ (Zymo Research, Irvine, CA, USA) to remove DNA molecules below 2 kb (following the instructions of the manufacturer). Viral DNA libraries were generated using the Nextera XT DNA Library Preparation Kit (Illumina, San Diego, CA, USA) following the instructions of the manufacturer. Tagged libraries were sequenced as part of a flow cell of 2 × 150 bp pair-ended NextSeq 550 (Illumina, San Diego, CA, USA) sequencing run.

### 2.4. Viral-Operational Taxonomic Unit (vOTU) Table

Following assembly and quality control (see Supplementary Methods), high-quality de-replicated reads from all samples were merged and recruited against all the assembled contigs at 95% similarity using Subread [34] and a contingency-table of reads per Kbp of contig sequence per million reads sample (RPKM) was generated forming the viral-operational taxonomic unit (vOTU)-table. The vOTU taxonomy was determined by querying the viral contigs against a database containing taxon signature genes for virus orthologous group hosted at www.vogdb.org using USEARCH-ublast (e-value 10^−3^). The set of vOTUs assigned eukaryotic viral taxonomy was considered the eukaryotic viral component. The sequences are available at the European Nucleotide Archive (ENA), http://www.ebi.ac.uk/ena/data/view/PRJEB34460.

### 2.5. Community Analysis

Prior to the bioinformatics analysis, vOTU’s that did not have a relative abundance above 0.5% in at least two samples were discarded. The sum abundance of removed vOTU’s constituted 3% or less per sample. vOTUs shorter than 3 kb were removed. Cumulative sum scaling [35] normalisation was performed using Quantitative Insight Into Microbial Ecology 1.9.1 [36] (QIIME 1.9.1). QIIME 2 (2018.4 build 1525276946) [36] was used for the subsequent analysis steps of alpha and beta diversity statistics. The Shannon, Simpson, and Richness alpha-diversity and Bray–Curtis dissimilarity index, Jaccard index and Sørensen–Dice coefficient beta diversity matrices were calculated. The Wilcoxon Rank Sum Test was used to evaluate the pairwise taxonomic differences and analysis of similarities (ANOSIM), and the Kruskal–Wallis tests was performed for group comparisons. For the presence/absence of key bacterial species, the qPCR threshold measured by Haahr et al. was used [31]. The k-mer based host-phage prediction algorithm WiSH (1.0) [37] was used to predict the prokaryotic hosts of viral features, based on vOTU sequences using closest common ancestor in cases of multiple high-confidence predictions. The WiSH host prediction models were based on 9747 bacterial genomes and 1970 phage genomes derived from NCBI RefSeq [38], September 2018.

The integrase content was estimated by mapping all viral reads/samples against a database of 247.000 viral integrase and transposase genes UniProt [39], September 2018 followed by calculation of the fraction of mapping reads. This was done to avoid bias caused by incomplete viral genome assembly. Each viral genome is unlikely to contain more than one integrase gene copy [40]. A temperate phage genome with fractured assembly would therefore appear as several vOTUs, with only one containing an integrase and be counted as one temperate and several lytic phages, which is circumvented using the approach outlined above.

Associations between the bacterial and viral community were performed using the mixOmics [41] R package using CSS-normalized operational taxonomic unit (OTU)-tables as input. Regularized canonical correlation analysis (rCCA) and sparse partial least squares (sPLS) were used to perform combined integration and variable selection on the viral and bacterial OTU-tables. The component tuning cross-validation procedures were double-checked using the shrinkage method for rCCA and leave-one-out cross validation for sPLS.

### 2.6. Ethics Statement

The study was approved by the regional ethical committee of the Central Denmark Region (file number 1-10-72-325-13) and the Danish Data Protection Agency (file number 1-16-02-26-14). The project was pre-registered at clinicaltrials.gov (file number NCT02042352).

## 3. Results

### 3.1. Samples and Sequencing

Purified viral particles from vaginal swabs (*n* = 48) were whole-genome sequenced, generating a total of 2,674,574 reads (a median of 44,868 paired end reads per sample) after joining, trimming, quality control, and discarding human and bacterial reads. A total of 773 viral operational taxonomic units (vOTUs) were retained after de novo assembly and filtering, sized from 3 to 85 kb with a median of 7.5 kb in length. The basic patient characteristics are shown in Table 1.

### 3.2. Composition of the Vaginal Virome

**Figure 1:**
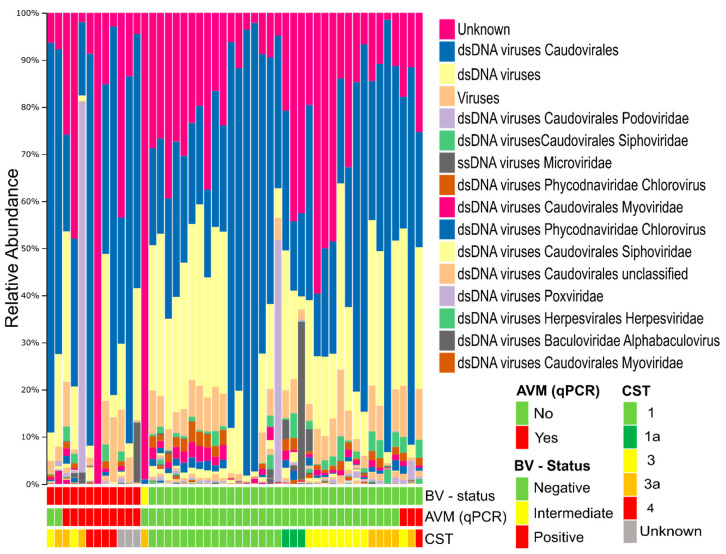
Composition of the vaginal virome by bacterial community status. Viral community composition by relative abundance, grouped by bacterial vaginosis (BV), abnormal vaginal microbiota (AVM), and the community state type (CST) of the sample bacterial community. Taxonomy based on viral database sequence match. Taxonomic entities are ordered top to bottom from most to least abundant.

A total of 61% of the de novo constructed contigs were identified as viral by matching to viral sequence databases. The remaining 39% of vOTUs had no matches in viral databases, but neither did they match bacterial sequences nor human DNA, which could be due to the detection of previously unidentified viruses. Viral matches were predominantly from the class of double stranded DNA (dsDNA) viruses, unidentified viruses, and a small proportion of single stranded DNA (ssDNA) viruses (Figure 1, Figure S1A). The investigated samples were previously classified as either BV-positive, BV-negative, or intermediate based on Nugent scoring of the bacteria in a Gram-stained smear [31]. There was no significant difference in the viral alpha diversity between BV-positive and BV-negative samples as determined by the Shannon diversity index and number of observed vOTUs (Figure S1B,C). Similarly, no correlation was found between the viral and bacterial alpha diversity (Figure S1D). Eukaryotic viruses were identified in all samples, constituting, on average, 4% of the total reads (Figure S2A).

### 3.3. Viral Beta Diversity is Strongly Correlated with BV-Status

**Figure 2:**
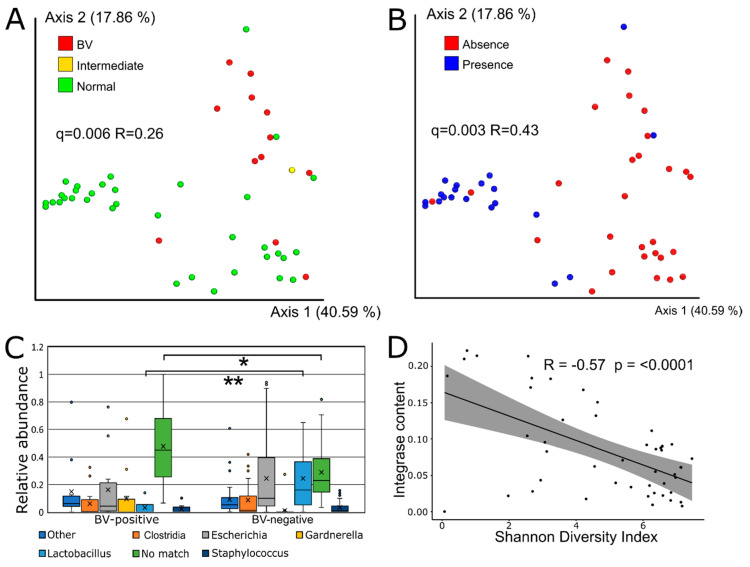
The vaginal viral community was significantly correlated with the BV-status. (**A**) Vaginal virome composition (Bray Curtis dissimilarity metric) by BV-status and (**B**) *Lactobacillus crispatus* presence/absence (determined by qPCR). (**C**) The relative abundance of WISH host genus predictions of vOTUs by BV status. The significance was calculated using the Kruskall–Wallis Test (* *p* < 0.05, ** *p* < 10^−3^). (**D**) A scatter plot showing the Shannon diversity against the percentage of raw reads mapping to integrase genes by sample. Significance levels were calculated using the Pearson correlation.

The viral community composition (as determined by Bray–Curtis dissimilarity metrics) was significantly different between BV-positive and BV-negative women (*q* = 0.006, *R* = 0.26) (Figure 2A). Comparison with a re-analysis of previously published data [28] on the bacterial community of the same samples (based on 16S rRNA gene amplicon data) showed that the vaginal virome composition was as strong a predictor of BV (Nugent score-based diagnosis) as the bacterial composition (*q* = 0.003, *R* = 0.50) (Figure S3A). qPCR-based quantification of key bacteria was previously shown to be a more accurate predictor of BV than traditional Nugent scoring [42]. In accordance, we found that the virome composition varied strongly with the qPCR determined presence/absence of *L. crispatus* (Figure 2B) and *L. iners* as well as the BV-associated *Gardnerella vaginalis* and *Atopobiom vaginae* (Figure S3B). The presence of *L. gasseri* and *L. jensenii* were not significantly associated with the viral component. Interestingly, the composition of the eukaryotic viral community also varied with the BV-status (*q* = 0.018, *R* = 0.20) (Figure S2B).

### 3.4. Sequence-Based Host Prediction and Integrase Content

Using WISH [37] to predict the bacterial host of viral OTUs showed that *Clostridium* was the most commonly predicted host genus, followed by *Lactobacillus* and *Gardnerella* (Figure S4A). BV-negative samples contained a significantly higher relative abundance of vOTUs predicted to have *Lactobacillus* spp. as their most likely host (*p* < 0.0001), while BV-positive samples contained significantly more vOTUs with no high-confidence host match (*p* = 0.03) (Figure 2C).

Lysogenic phages can be identified using marker genes, such as integrases, which are specific to lysogenic phages [43]. The ratio between lytic and lysogenic phages was estimated by comparing the integrase gene content between samples. There was no statistically significant difference in the fraction of reads mapping to integrase/transposase sequences between samples with different BV-statuses, although BV-negative samples had a higher mean fraction (0.091 ± 0.004) compared to BV-positive samples (0.066 ± 0.005) (Figure S4B). There was a highly significant negative correlation between the viral alpha diversity (Shannon diversity index) and the fraction of viral reads matching integrase genes in each sample (*p*-value < 0.0001) (Figure 2D).

### 3.5. Bacteriophage-bacteria Interactions

**Figure 3:**
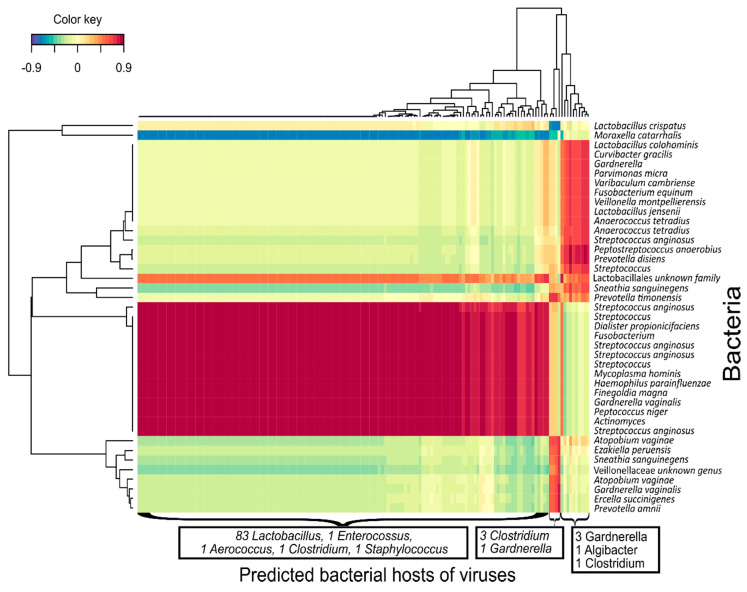
Abundance of phages with beneficial hosts correlated negatively with an abundance of pathogenic bacteria and vice versa. The clustered image map (CIM) of regularized canonical correlation analysis (rCCA) between the relative abundances of bacterial and viral operational taxonomic units (vOTUs). The colour grade shows the strength of correlation between individual bacterial and viral OTUs. Viral clusters were summarized based on their bacterial host genus as predicted by WISH as only a minority had matches in the viral databases. Bacterial OTUs with several entries had distinct 16S sequences and were possibly different strains. Correlations above 0.7 are shown. CV-score = 0.82.

Co-abundance analysis of bacterial OTUs and vOTUs was performed using regularized extension of canonical correlation analysis (rCCA) to reveal correlations between individual phages and bacteria. The rCCA analysis was separately performed for BV positive and negative samples under the assumption that the microbial dynamics and environmental conditions, such as the pH, would be highly different between the two groups. The correlation analysis revealed large sets of strongly correlated bacterial OTUs and vOTUs as shown for the BV-positive samples in Figure 3. When comparing the cross validation (CV) scores, BV-positive samples had strong correlations between bacterial and viral OTUs (CV-score 0.82) in comparison to BV-negative samples (CV-score 0.38) (Figure S5A) and all 48 samples together (CV-score 0.36) (Figure S5B).

## 4. Discussion

### 4.1. Main Findings

This study provides a novel in-depth characterization of the vaginal DNA virome based on virus-like particle purification followed by metagenomic sequencing and de novo assembly, constructing a total of 773 vOTUs (of these 302 represent previously undescribed viruses/phages). We found that the composition of both the prokaryotic and the eukaryotic viral communities varied strongly between BV-negative and BV-positive samples. Clear co-abundance patterns between certain bacteria and vOTUs indicated that these two components of the vaginal microbiome were strongly interlinked. The eukaryotic viral component differed significantly between BV-positive and negative samples, although these viruses are not directly interacting with the bacterial community. The identified vOTUs were predominantly dsDNA viruses, unidentified viruses, and a small proportion of ssDNA viruses; however, only a minority had exact matches in viral databases. This is not unexpected considering the lack of previous characterization of the vaginal virome using de novo assembly and an overall underrepresentation of viral genomes in relevant databases [24].

Co-abundance correlation showed that groups of mainly anaerobic bacteria, including *Gardnerella* spp. and *Moraxella catarrhalis,* were negatively correlated with a large number of vOTUs predicted to target *Lactobacillus* spp. and other commensal bacteria. This likely reflects that vaginal microbiota that contain many lactobacilli have low abundances of BV-associated bacteria and, therefore, also lack the corresponding phages. In this aspect, the finding of distinct viral groups could indicate the presence of distinct vaginal viral communities that correspond to the bacterial composition. However, larger studies are needed to determine whether such viral communities of distinct composition, or viral CSTs, are consistently found in the VMB and whether they are tied to bacterial CSTs [2]. The viral community structure was strongly correlated with the presence (determined by qPCR thresholds) of key beneficial bacterial species (*L. crispatus* and *L. iners*) as well as with known pathobionts (*Gardnerella* spp. and *A. vaginae*). This demonstrates a clear link between the viral component and key bacterial indicators of vaginal microbiome health and dysbiosis.

The observation that the eukaryotic viral community differed by BV-status was surprising as these are not directly linked to the bacterial community. We speculate that rather than being directly linked to the dysbiotic bacterial community associated with BV, the differences in the eukaryotic viral community composition reflect environmental factors, such as low pH, which is linked with healthy microbiota status and has been shown to inhibit viral infectivity and survival [44]. In line with this, a *Lactobacillus crispatus*-dominated cervicovaginal microbiota was previously associated with reduced genital HIV viral load and HIV/STI prevalence [9]. Of the identified eukaryotic virus DNA, the *Herpesvirales* and *Pappilomaviridae* orders were the only groups currently known to be associated with human disease.

### 4.2. Biological Role of the Virome and Phages in Vaginal Health and Disease

Given the observation that large groups of bacteria and bacteriophages correlated in a biologically meaningful fashion, it is likely that bacteriophages play a role in shaping the vaginal bacterial community. Expansions of the viral community appear to be caused by externally originating lytic phages rather than the activation of prophage elements from bacterial genomes as samples with highly diverse viral communities contain a smaller ratio of lysogenic phages. The reason for this could be that a more diverse and unstable bacterial composition favours the lytic lifestyle in the corresponding virome and that a simple, stable community favours temperate phages. This is supported by computational models showing how the virulent strategy works best for phages with a large diversity of hosts and with access to multiple independent environments reachable by diffusion [45]. Therefore, the vaginal mucosa, which is perturbated by multiple factors, such as intercourse, female hygiene products, and menstruation [46], could favour lytic replication in comparison to the more sequestered intestinal mucosa where lysogeny dominates [47].

There are several possible mechanisms of phage-related vaginal dysbiosis. One is the introduction of foreign phages (i.e., from the partner) depleting the commensal bacteria and allowing pathogen colonization. Alternatively, pathogenic bacteria could be directly colonizing with their corresponding phages as passengers. In the first case, vaginal stability would require commensal bacteria to be resistant to invading phages, for example by containing a prophage element of a related phage, providing immunity in the form of superinfection exclusion [48]. In the scenario of direct pathogen colonization, phages in the vaginal mucosa could protect against these, thereby providing non-host derived immunity. Women lacking these protective phages in the vaginal mucosa would then be more susceptible to develop dysbiosis. In this case, phage-therapy with a cocktail of phages could be used to target vaginal pathogens specifically, allowing the commensal bacterial population to re-establish. From this study, it is not possible to determine whether bacterial dysbiosis preceded or followed changes in the viral community. Studies using longitudinal vaginal samples are needed to elucidate the temporal dynamics of these two communities in detail.

### 4.3. Comparisons to Other Studies

Our findings contrast with the previous findings of Gosmann et al. [25], where no distinct viral community structure differences between BV-positive and negative samples were found. However, contrary to Gossman et al., the present study up-concentrated VLPs prior to sequencing and used a de novo construction approach, allowing the detection of low-abundant viral sequences not already present in databases, improving the sensitivity. The filtration of VLPs before sequencing was shown to increase sensitivity, and is recommended for human virome studies [49]. Gosmann et al. extracted viral DNA from only 300 µL of cervico-vaginal lavage (a 40-fold dilution [50]) and only analyzed viral reads that had a high similarity to database matches. However, human virome databases are still very scarce and up to 80% of our viral contigs did not have matches in viral databases even at the family level, although the contigs harbored many phage-associated genes, such as capsid-encoding genes, integrases etc. We, therefore, conclude that our approach was more sensitive, especially considering that the databases Gossman et al. used were accessed in 2017 and, therefore, even less extensive.

### 4.4. Limitations

Viral metagenome sequencing measures the relative and not the absolute abundance; thus, it is possible that there could be differences in the overall viral load that were not detected. The relatively small sample size and lack of longitudinal samples limits the advanced analysis of phage-bacteria dynamics. The limited detection of negatively correlated phage-host pairs is likely due to the lack of longitudinal sampling, as murine enteric studies have shown that the increase in phage abundance and host decrease occurs over a 7–8 day period before reaching new stable levels [51]. This study only investigated the DNA virus fraction of the vaginal virome, and RNA viruses, which includes many clinically relevant viruses, were thus not included in the study. While RNA bacteriophages are relatively scarce, and not thought to play a major role in the human microbiome [52], they represent an area in need of further study.

## 5. Conclusions

In conclusion, this first, in-depth investigation of the vaginal DNA virome determined that the vaginal viral community is strongly correlated with the vaginal bacterial community and BV. Therefore, including the viral component has the potential to provide a more complete understanding of the mechanisms in vaginal microbial health and dysbiosis. What remains to be determined is the strength and, of equal importance, the direction of the interaction between the vaginal viral and bacterial component, which will require longitudinal studies.

## Figures and Tables

**Table 1:** Overview of the patient characteristics.

	Patients, No. (%) ^a^ (*n* = 48)
Age, median (range), y	29 (23–41)
Body mass index, median(range) ^b^	31 (17.5–41)
Ethnicity	
Caucasian	83 (40)
Eastern European	8 (4)
Other	6 (3)
Asian	2 (1)
Cause of infertility	
Male factor	58 (28)
Single	13 (6)
Lesbian	3 (2)
Nugent Group ^c^	
BV	25 (12)
Normal	73 (35)
Intermediate	2 (1)

^a^ Data represent the No. (%) of patients unless otherwise specified. ^b^ The body mass index was calculated as the weight in kilograms divided by height in meters squared. ^c^ Based on Nugent scoring of Gram-stained smears.

## Supplementary Materials

The following are available online at https://www.mdpi.com/1999-4915/12/10/1143/s1, Figure S1A: The relative viral abundance summarized at the group level, Figure S2B: The viral alpha diversity by Shannon diversity index, Figure S2C: The viral alpha diversity by observed OTUs, Figure 2D: Boxplot of viral vs. bacterial alpha diversity by the Shannon diversity index, Figure S2A: The relative abundance of eukaryotic viral OTUs, Figure S2B: PCoA plot of Bray–Curtis dissimilarity beta diversity by BV-status, Figure S3A: PCoA plot of Bray–Curtis dissimilarity beta diversity bacterial component Figure S3B: Bray–Curtis dissimilarity by the presence/absence of key bacterial species, Figure S4A: WISH host predictions of vOTUs, Figure S5A: Clustered image map (CIM) of regularized canonical correlation analysis (rCCA) between the relative abundances of bacterial OTU and viral OTUs of all samples, Figure S5B: Clustered image map (CIM) of regularized canonical correlation analysis (rCCA) between relative abundances of bacterial OTU and viral OTUs of BV-negative samples, Figure S6: Trimming, cleaning, assembly, and deduplication of VLP-derived metagenome reads. Supplementary Methods.

## Clinical Trials Registration

NCT02042352.
