# Supplementary material for: Characterization of the Vaginal DNA Virome in Health and Dysbiosis
Source: Viruses. 2020 Oct 9;12(10):1143. doi: 10.3390/v12101143 (PMC7600586; doi:10.3390/v12101143)
Supplement: Supplementary file 1 [file viruses-12-01143-s001.pdf]

viruses

MDPI

Supplementary materials

Figure S1. Vaginal viruses: taxonomy, alpha and beta-diversity

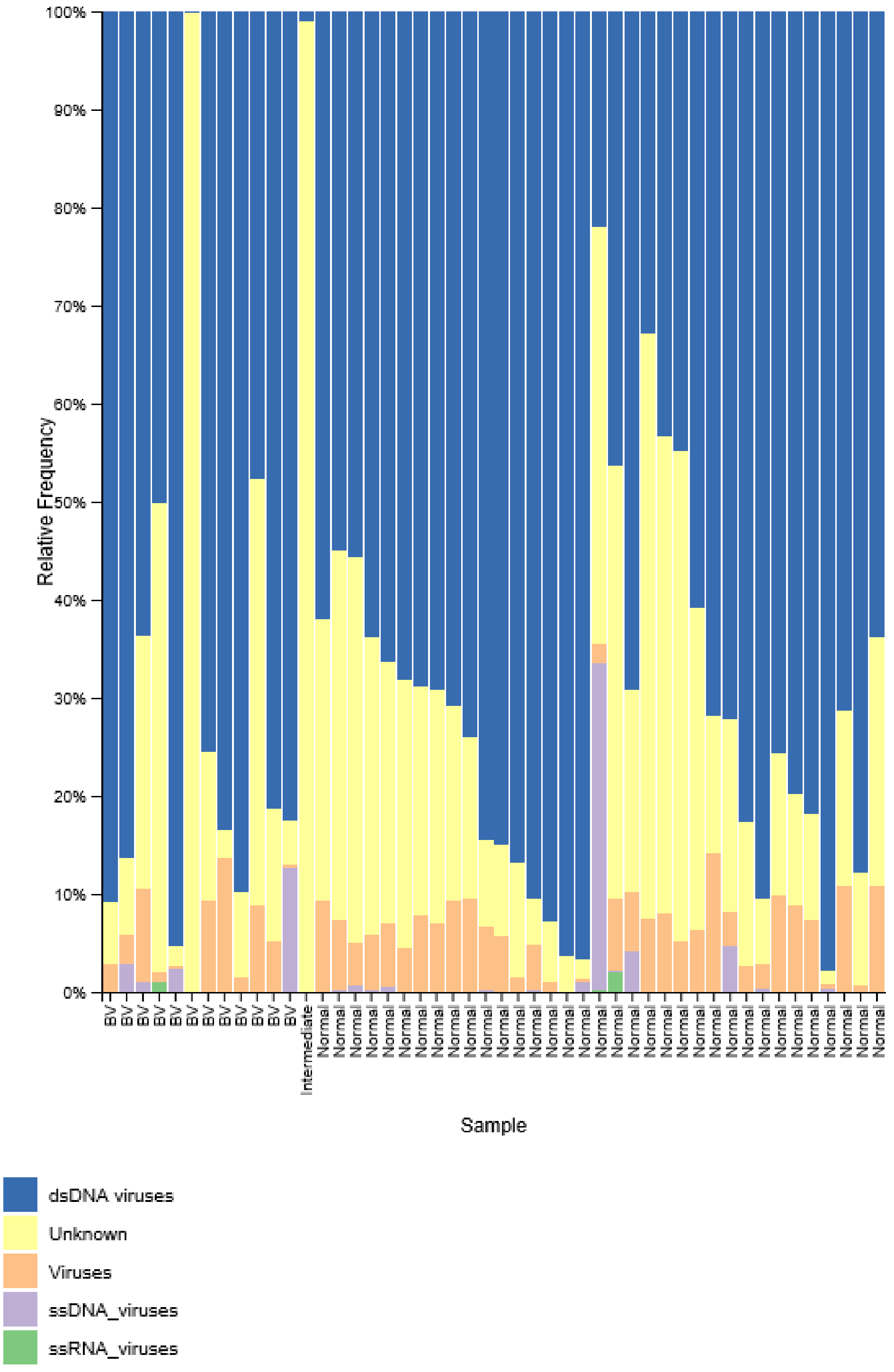

Figure S1A. Relative viral abundance summarized at group level. Taxonomy based on closest viral database hit. Samples grouped by BV-status as determined by nugent scoring.

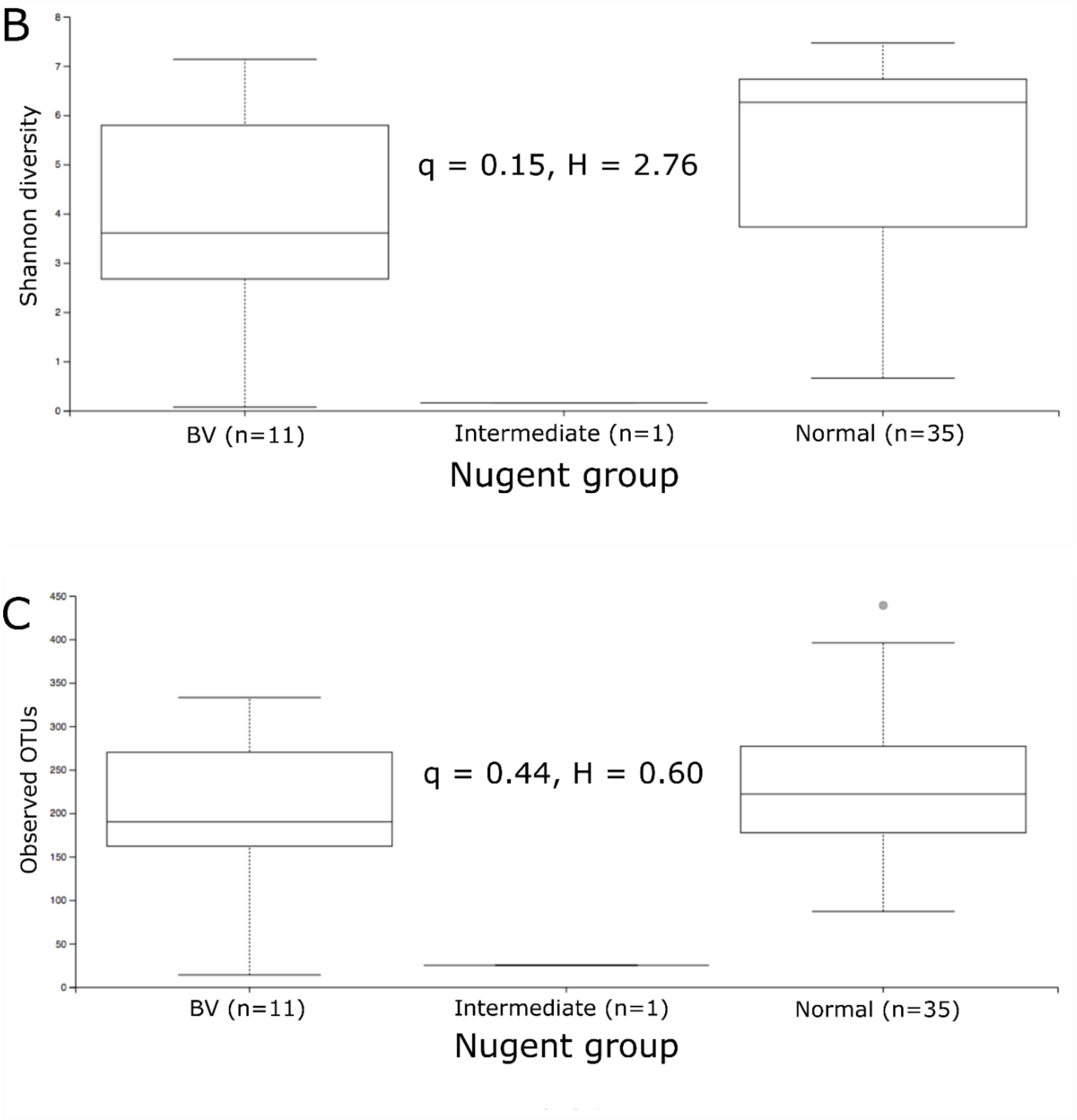

Figure S1B. Viral alpha diversity by (**B**) Shannon diversity index and (**C**) Observed OTUs. No significant correlations found by Pairwise Kruskall-Wallis test.

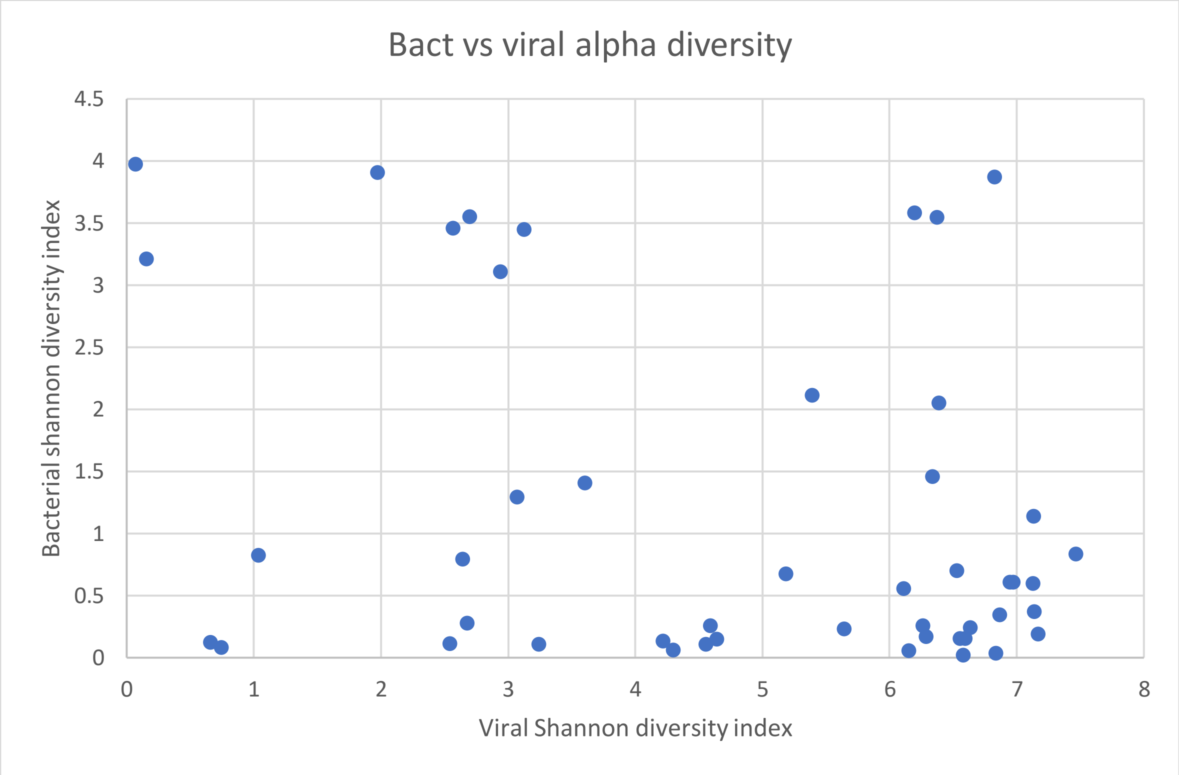

Figure S1D. Bacterial vs viral alpha diversity. Scatterplot showing viral vs bacterial alpha diversity by Shannon diversity index for each sample. No significant correlation was found.

Figure S2. Vaginal Eukaryotic viruses

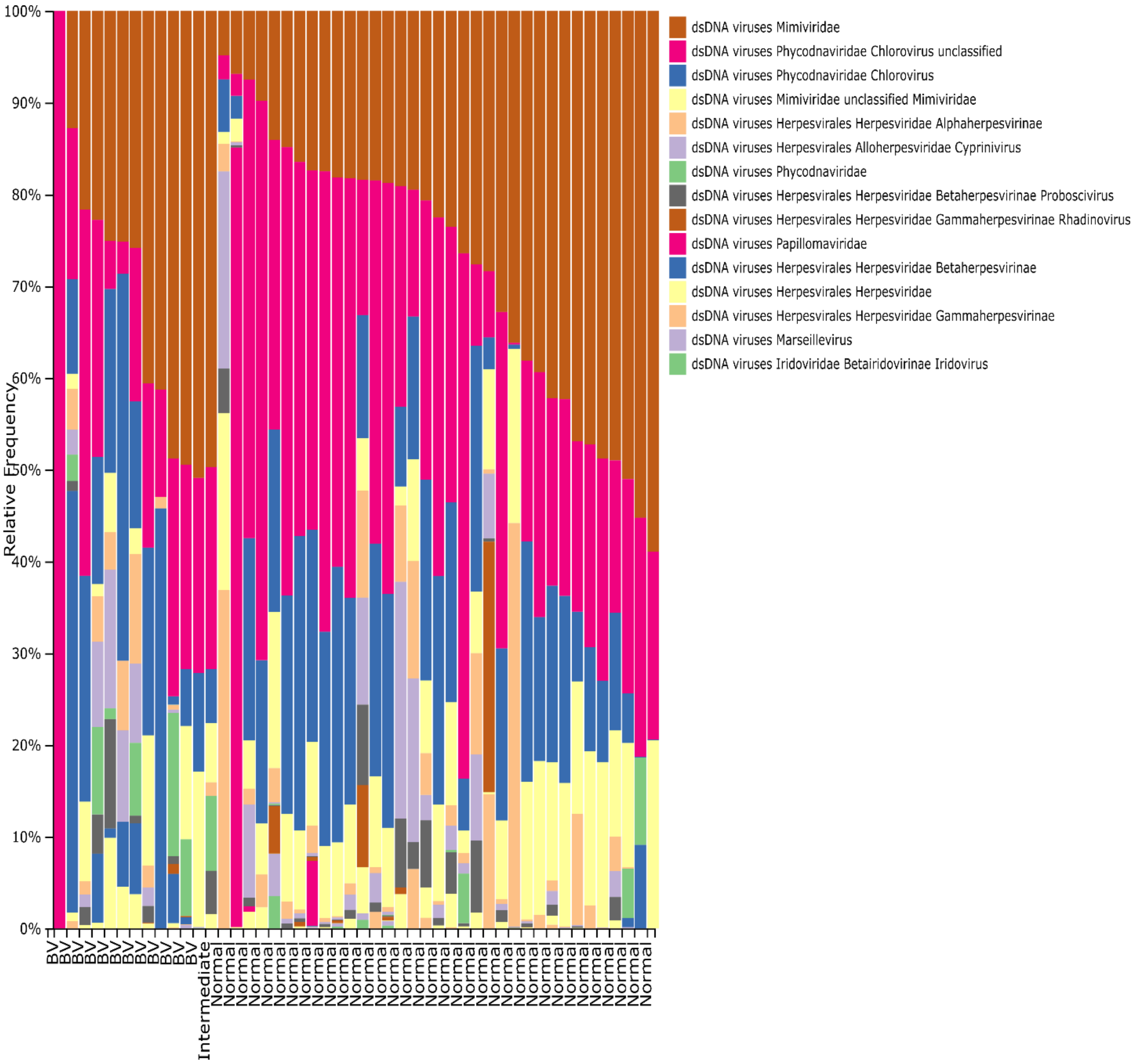

Figure S2A. Relative eukaryotic viral abundance at highest possible level of taxonomic identification. Only vOTUs with eukaryotic viral database matches were included. Taxonomic identification to highest high confidence match. All viruses classified as eukaryotic were extracted from the OTU-table and analysed as a separate subset.

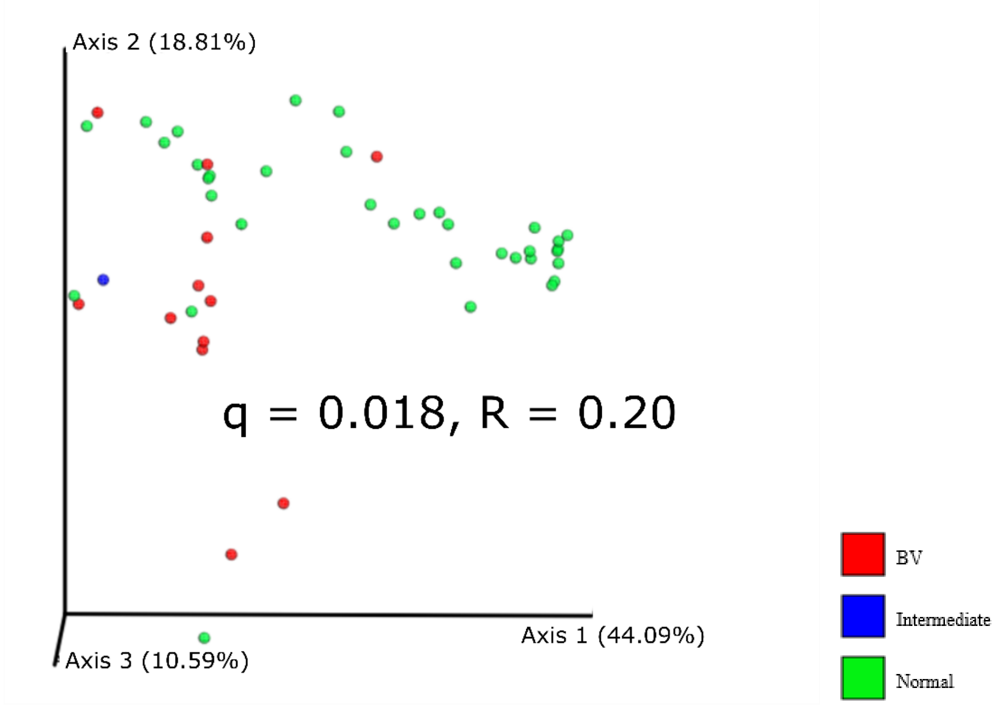

Figure S2B. Eukaryotic viral beta diversity. PCoA plot of Bray-Curtis dissimilarity beta diversity by BV-status. Group comparison by pairwise ANOSIM. All viruses classified as eukaryotic were extracted from the OTU-table and analysed as a separate subset.

Figure S3. Vaginal bacterial component

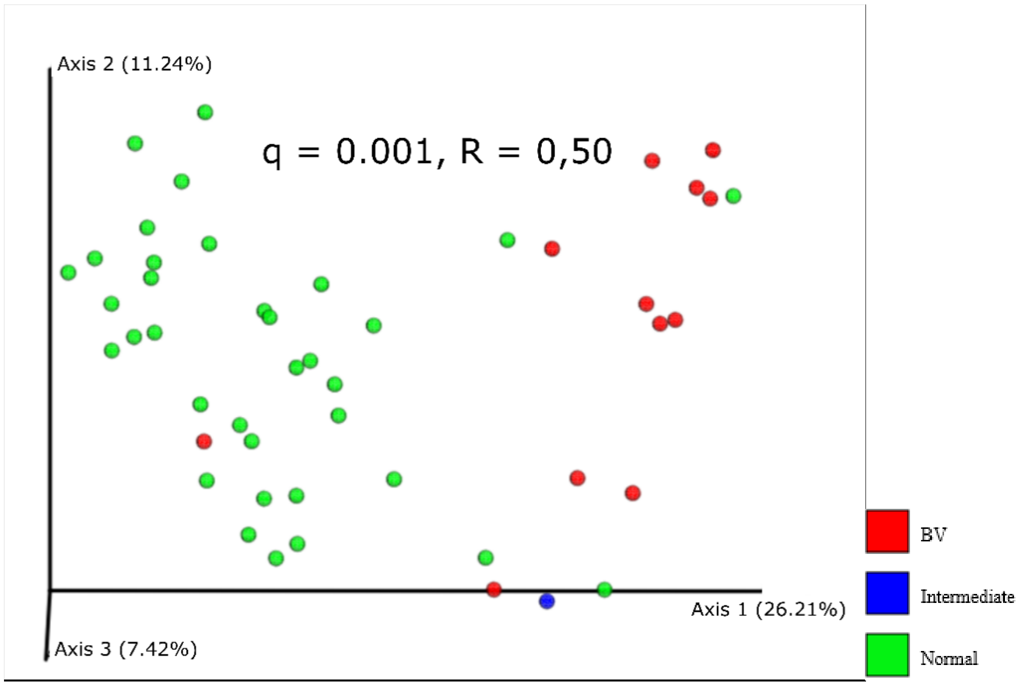

Figure S3A. Bacterial beta diversity. PCoA of Bray-Curtis dissimilarity beta diversity of vaginal bacterial component by BV-status. Group comparison by pairwise ANOSIM.

Bacteria	*R*	*p*-value
Lactobacillus crispatus	0.43069	0.001
Lactobacillus iners	0.148474	0.002
Lactobacillus jensenii	-0.10973	0.987
Lactobacillus gasseri	-0.114594	0.962
Gardnerella vaginalis	0.209314	0.003
Atopobium vaginae	0.247796	0.002

Figure S3B. Viral community correlations presence/absence of key bacteria. Bray-Curtis dissimilarity by presence/absence of key bacterial species as determined by qPCR. ANOSIM pairwise comparisons.

Figure S4. Viral-bacterial correlations

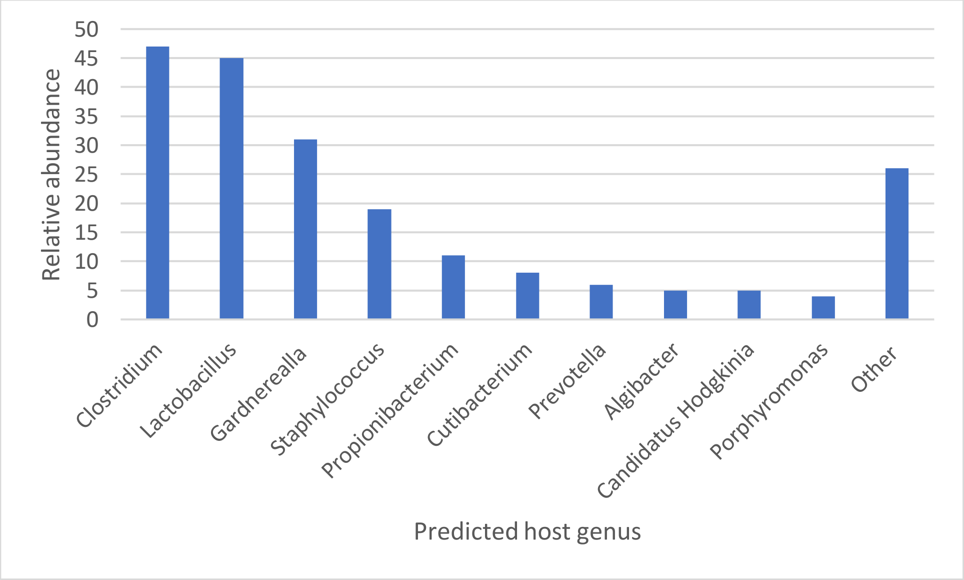

Figure S4A. Viral host predictions. WIsH viral host prediction of vOTUs across all samples by relative abundance, grouped at genus level.

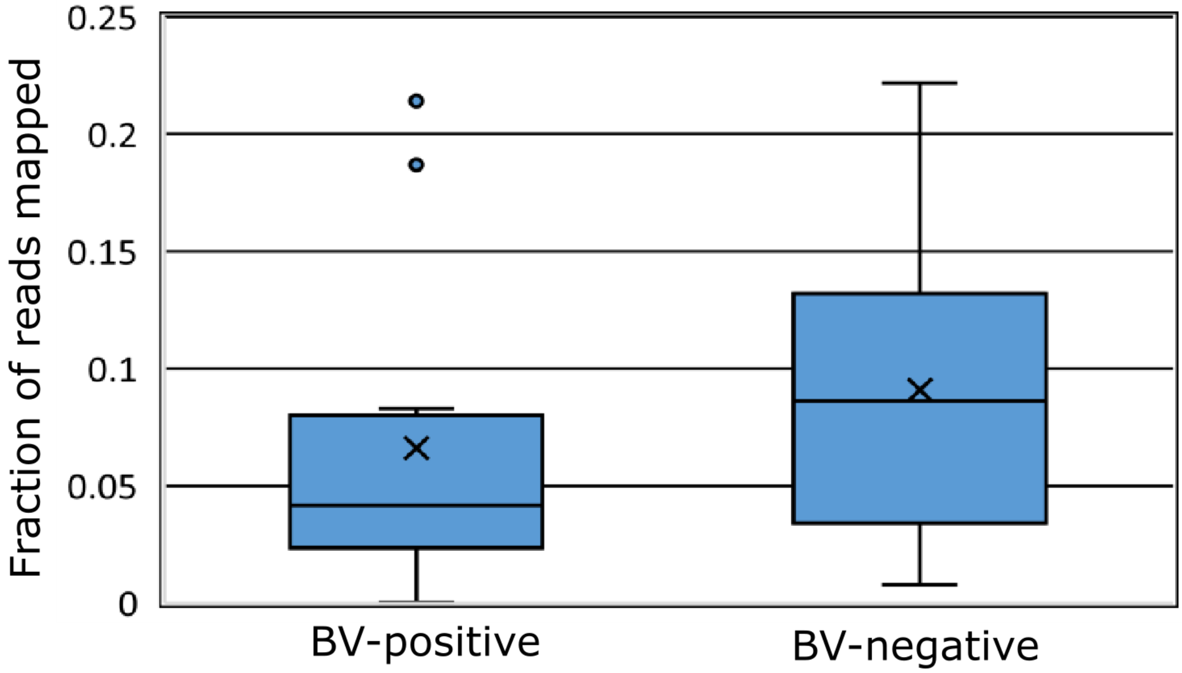

Figure S4B. Viral integrase content by BV-status. Integrase content of viral component by BV status. Percentage of reads mapping integrase genes. No significant difference.

Figure S5. Viral-bacterial correlation: Regularized extension of the Canonical Correlation Analysis

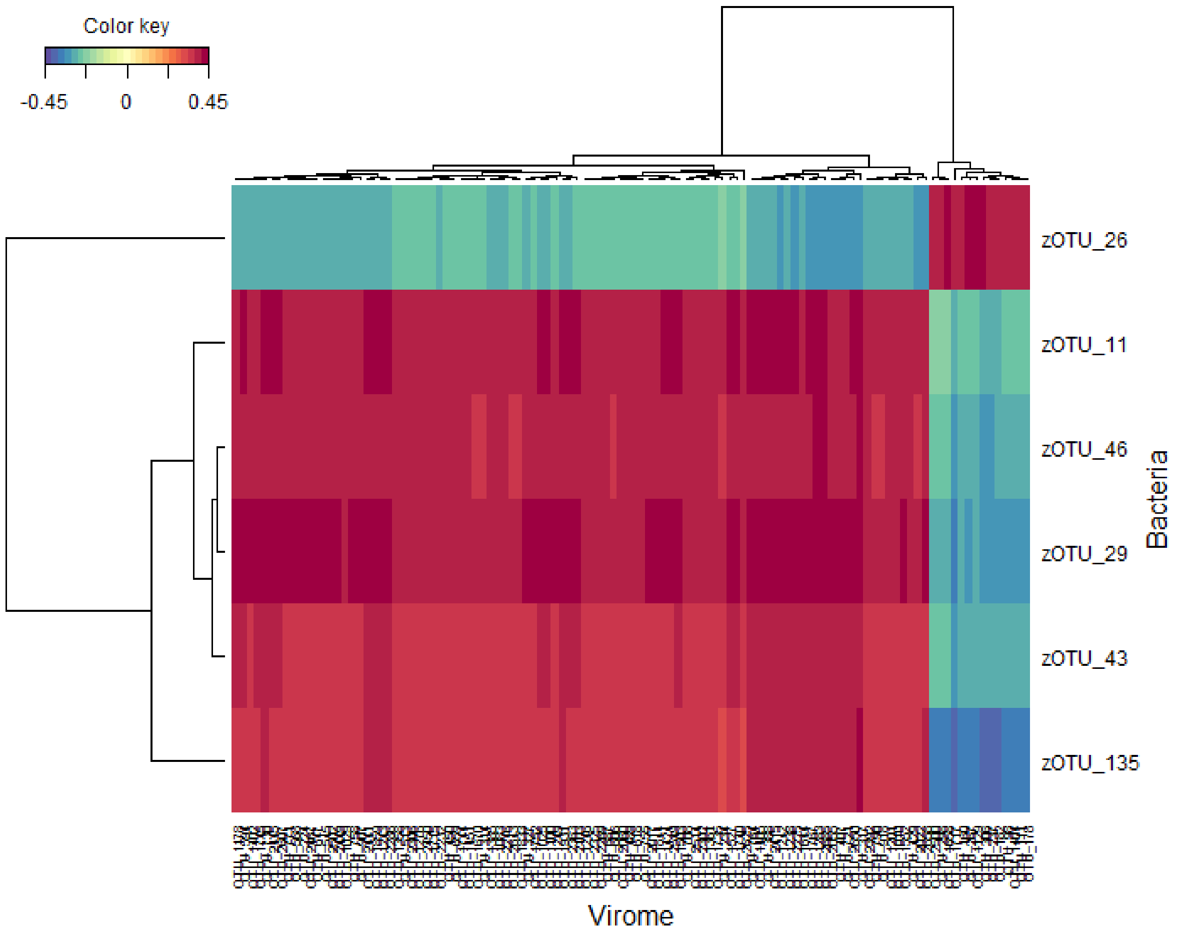

Figure S5A. Bacterial-viral corralations for BV-negative samples. Clustered Image Map (CIM) of regularized Canonical Correlation Analysis (rCCA) between relative abundances of bacterial OTU and viral OTUs of all samples. Colour grade shows strength of correlation between individual bacterial and vOTUs. Viral clusters were summarized based on their bacterial host genus as predicted by WIsH as only a minority had matches in viral databases. Bacterial OTUs with several entries had distinct 16S sequences and are possibly different strains. Correlations above 0.4 are shown. CV-score = 0.36.

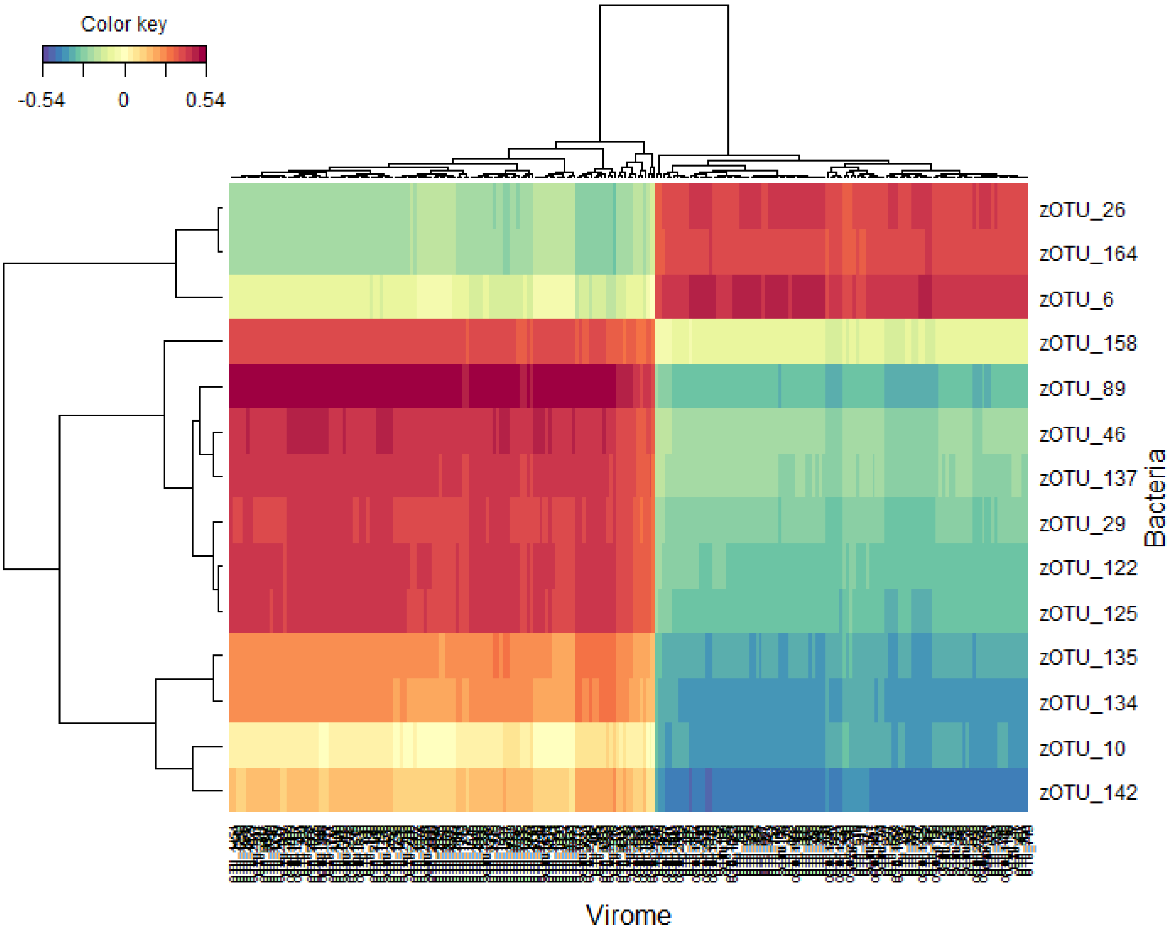

Figure S5B. Bacterial-viral corralations for BV-negative samples. Clustered Image Map (CIM) of regularized Canonical Correlation Analysis (rCCA) between relative abundances of bacterial OTU and viral OTUs of BV-negative samples. Colour grade shows strength of correlation between individual bacterial and vOTUs. Viral clusters were summarized based on their bacterial host genus as predicted by WIsH as only a minority had matches in viral databases. Bacterial OTUs with several entries had distinct 16S sequences and are possibly different strains. Correlations above 0.4 are shown. CV-score = 0.38.

Supplementary methods

Trimming, cleaning, assembly and deduplication of VLP-derived metagenome reads

The raw reads were trimmed from adaptors and barcodes using Trimmomatic v0.35[1] (>97% quality [seedMismatches: 2, palindromeClipThreshold: 30, simpleClipThreshold:10; LEADING: 15; MINLEN: 50], removed from ΦX174-control DNA and de-replicated (Usearch v10)[2]. Non-redundant high-quality reads with a minimum size of 50nt were retained. The presence of non-viral DNA was quantified using 50,000 random forward-reads from each sample, which were queried against the human genome, as well as all the bacterial and viral genomes hosted at NCBI using Kraken2 [3]. Similarly, reads were blasted against the non-redundant protein database available at UniProtKB/Swiss-Prot (-evalue 1e-3, -query_cov 0.6, -id 0.7), the ribosomal 16S rRNA (GreenGenes 13_5[4]) and 18S rRNA (Silva, release 126[5]) databases (-evalue 1e-3, -query_cov 0.97, -id 0.97).

Reads generated from VLP-derived DNA sequencing were subjected to within-sample de novo assembly. Assembly was carried out using metaSPAdes v3.5.0[6] and only scaffolds (here termed "contigs") with a minimum length of 1,000 nt were retained. Contigs generated from all samples were pooled and de-replicated by multiple blasting and removing those contained in over 90% of the length of another (90% similarity), as outlined previously[7]. To check the presence of non-viral DNA

contigs, de-replicated contigs were evaluated according to their match to a wide range of viral proteins, [viral non-redundant RefSeq [8], virus orthologous proteins (www.vogdb.org), and the prophage/virus database hosted at PHASTER (www.phaster.ca[9])], reference independent k-mer signatures [VirFinder[10]], viral genomes RefSeq [NCBI, Kraken2] as well as their match to bacterial (--confidence 0.08), plant (Kraken2, --confidence 0.3) and human genomes (Kraken2, --confidence 0.1) deposited in the NCBI database. All contigs that did not match any database or that matched viral sequences, viral proteins or viral k-mers were subsequently retained and categorized as viral contigs.

References

1. Bolger AM, Lohse M, Usadel B. Trimmomatic: a flexible trimmer for Illumina sequence data. Bioinformatics. Oxford University Press; **2014**; 30(15):2114–20.
2. Edgar RC. UPARSE: Highly accurate OTU sequences from microbial amplicon reads. Nat Methods. Nature Publishing Group; **2013**; 10(10):996–998.
3. Wood DE, Salzberg SL. Kraken: ultrafast metagenomic sequence classification using exact alignments. Genome Biol. **2014**; 15(3):R46.
4. McDonald D, Price MN, Goodrich J, et al. An improved Greengenes taxonomy with explicit ranks for ecological and evolutionary analyses of bacteria and archaea. ISME J. Nature Publishing Group; **2012**; 6(3):610–8.
5. Quast C, Pruesse E, Yilmaz P, et al. The SILVA ribosomal RNA gene database project: improved data processing and web-based tools. Nucleic Acids Res. Oxford University Press; **2013**; 41(Database issue):D590-6.
6. Nurk S, Meleshko D, Korobeynikov A, Pevzner PA. metaSPAdes: a new versatile metagenomic assembler. Genome Res. Cold Spring Harbor Laboratory Press; **2017**; 27(5):824–834.
7. Reyes A, Blanton L V, Cao S, et al. Gut DNA viromes of Malawian twins discordant for severe acute malnutrition. Proc Natl Acad Sci U S A. National Academy of Sciences; **2015**; 112(38):11941–6.
8. Liao Y, Smyth GK, Shi W. The Subread aligner: fast, accurate and scalable read mapping by seed-and-vote. Nucleic Acids Res. Oxford University Press; **2013**; 41(10):e108.
9. Arndt D, Grant JR, Marcu A, et al. PHASTER: a better, faster version of the PHAST phage search tool. Nucleic Acids Res. **2016**; 44(May):1–6.
10. Ren J, Ahlgren NA, Lu YY, Fuhrman JA, Sun F. VirFinder: a novel k-mer based tool for identifying viral sequences from assembled metagenomic data. Microbiome. BioMed Central; **2017**; 5(1):69.
